# Targeting Ocular Biofilms with Plant-Derived Antimicrobials in the Era of Antibiotic Resistance

**DOI:** 10.3390/molecules30132863

**Published:** 2025-07-05

**Authors:** Monika Dzięgielewska, Michał Tomczyk, Adrian Wiater, Aleksandra Woytoń, Adam Junka

**Affiliations:** 1 Platform for Unique Models Application (P.U.M.A.), Department of Pharmaceutical Microbiology and Parasitology, Wroclaw Medical University, ul. Borowska 211, 50-534 Wrocław, Polandaleksandra.woyton@student.umw.edu.pl (A.W.); 2 Dziegielewska Eye Institute, ul. Prymasa Augusta Hlonda 10c/u7, 02-972 Warsaw, Poland; 3 Department of Biology and Pharmacognosy, Faculty of Pharmacy with the Division of Laboratory Medicine, Medical University of Białystok, ul. Mickiewicza 2a, 15-230 Białystok, Poland; 4 Department of Industrial and Environmental Microbiology, Institute of Biological Sciences, Faculty of Biology and Biotechnology, Maria Curie-Skłodowska University, ul. Akademicka 19, 20-033 Lublin, Poland; adrian.wiater@mail.umcs.pl

**Keywords:** ophthalmic infections, biofilm, antimicrobial resistance, phytochemicals, phytotherapy

## Abstract

Microbial biofilms present a formidable challenge in ophthalmology. Their intrinsic resistance to antibiotics and evasion of host immune defenses significantly complicate treatments for ocular infections such as conjunctivitis, keratitis, blepharitis, and endophthalmitis. These infections are often caused by pathogens, including *Staphylococcus aureus*, *Pseudomonas aeruginosa*, and *Candida albicans*, particularly in patients using contact lenses or intraocular implants—devices that serve as surfaces for biofilm formation. The global rise in antimicrobial resistance has intensified the search for alternative treatment modalities. In this regard, plant-derived antimicrobials have emerged as promising candidates demonstrating broad-spectrum antimicrobial and antibiofilm activity through different mechanisms from those of conventional antibiotics. These mechanisms include inhibiting quorum sensing, disrupting established biofilm matrices, and interfering with microbial adhesion and communication. However, the clinical translation of phytochemicals faces significant barriers, including variability in chemical composition due to environmental and genetic factors, difficulties in standardization and reproducibility, poor water solubility and ocular bioavailability, and a lack of robust clinical trials evaluating their efficacy and safety in ophthalmic settings. Furthermore, regulatory uncertainties and the absence of unified guidelines for approving plant-derived formulations further hinder their integration into evidence-based ophthalmic practice. This review synthesizes the current knowledge on the pathogenesis and treatment of biofilm-associated ocular infections, critically evaluating plant-based antimicrobials as emerging therapeutic agents. Notably, resveratrol, curcumin, abietic acid, and selected essential oils demonstrated notable antibiofilm activity against *S. aureus*, *P. aeruginosa*, and *C. albicans*. These findings support the potential of phytochemicals as adjunctive or alternative agents in managing biofilm-associated ocular infections. By highlighting both their therapeutic promise and translational limitations, this review contributes to the ongoing discourse on sustainable, innovative approaches to managing antibiotic-resistant ocular infections.

## 1. Introduction

Across the globe, microbial infections remain one of the leading causes of ocular diseases. These infections may be mono- or polymicrobial in nature and are often linked to several predisposing factors, including ocular trauma, surgical procedures, advanced age, dry-eye syndrome, chronic nasolacrimal duct obstruction, contact lens use, and a history of prior ocular infections [1,2].

In parallel with the growing threat of antimicrobial resistance, scientific interest has increasingly turned toward natural compounds with therapeutic potential. Among these, plant-derived antimicrobials—such as polyphenols, essential oils, terpenoids, and alkaloids—have demonstrated significant antibacterial, antifungal, and antibiofilm properties in various models. Their multifaceted mechanisms of action, structural diversity, and relatively low toxicity make them attractive candidates for treating infections, particularly those involving biofilm formation. This emerging field holds promise for the development of novel adjunctive or alternative strategies in ophthalmology. A wide spectrum of bacterial pathogens is responsible for ocular conditions such as conjunctivitis, keratitis, endophthalmitis, blepharitis, orbital cellulitis, and dacryocystitis [3,4]. Among these conditions, conjunctivitis—inflammation of the conjunctival membrane—is the most common. While often self-limiting, it can create substantial clinical, economic, and social burden [5]. In its chronic form, inflammation may extend to adjacent structures, including the eyelids, thereby increasing the risk of intra- and extraocular complications. Bacteria account for 50–70% of conjunctivitis cases. These most frequently affect children and the elderly but are also reported in neonates and adults [6]. Blepharitis, defined as inflammation of the eyelid margins, may cause eyelash loss and extend into surrounding ocular structures, exacerbating infection [7,8]. In contrast, keratitis is a more severe condition that remains a major cause of corneal blindness. If not treated promptly, keratitis can progress to the vision-threatening intraocular infection, endophthalmitis. Endophthalmitis may be exogenous, following ocular trauma or surgery (e.g., cataract extraction), or endogenous, resulting from the hematogenous spread of systemic infection [3]. Dacryocystitis, which is typically associated with chronic obstruction of the nasolacrimal duct, results in tear stagnation, secondary bacterial colonization, and infection. If left untreated, this condition may lead to corneal damage, intraocular spread, and postoperative complications, including endophthalmitis [1,9]. In postoperative inflammatory conjunctivitis, Gram-positive bacteria are most often implicated, particularly *Staphylococcus aureus*, *S. epidermidis*, and *Streptococcus pyogenes*. Less frequently, infections may involve *Haemophilus influenzae*, *Moraxella lacunata*, *Chlamydia trachomatis*, *Pseudomonas aeruginosa*, and the fungus *Candida albicans*. Notably, methicillin-resistant *S. aureus* (MRSA) is increasingly recognized as a dominant ocular pathogen. It is associated with purulent conjunctivitis and eyelid and periocular soft tissue infections, especially among hospitalized ophthalmic surgery patients [9,10,11,12,13,14,15,16,17,18,19]. With MRSA colonization rates ranging from 20 to 70%, infection risk is significantly heightened; the complex resistance mechanisms of this pathogen make its eradication clinically challenging [20]. Contact lens-associated keratitis represents a distinct and significant clinical entity [21]. Infections may not only arise from microbiota colonizing the ocular surface and adnexa but also from biofilm-forming pathogens adhering to contact lenses. The most involved organisms are, again, Gram-positive cocci (*Staphylococcus* spp.), but also Gram-negative bacilli, particularly *P. aeruginosa* [22]. Although *P. aeruginosa* is not part of the normal ocular microbiota, the abiotic surface of contact lenses offers an ideal environment for adherence and proliferation.

**Figure 1:**
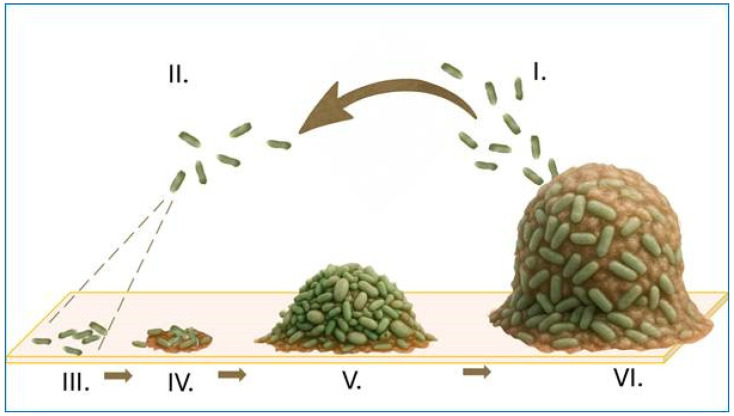
Stages of biofilm formation. Biofilm development is a cyclical and regulated process initiated by the dispersal of bacterial cells from a pre-existing mature biofilm (**I**). These free-floating planktonic cells (**II**) actively migrate through the surrounding environment in search of favorable conditions. Upon encountering a solid surface (represented by the horizontal rectangle), they undergo reversible and then irreversible attachment (**III**), mediated by adhesins, surface appendages, and physicochemical interactions. This leads to the formation of early microcolonies (**IV**), accompanied by clonal expansion, quorum sensing activation, and the initial secretion of extracellular polymeric substances (EPS). As the structure matures (**V**), cells become embedded in a dense matrix composed of polysaccharides, proteins, lipids, and extracellular DNA. The resulting mature biofilm (**VI**) is a highly structured and resilient community exhibiting spatial heterogeneity, metabolic cooperation, and increased tolerance to antimicrobials. The cycle continues as a subpopulation of cells undergoes regulated dispersal.

The above-mentioned microorganisms exist in the form of biofilms—structured communities encased in a self-produced matrix—on abiotic (e.g., contact lenses) or biotic (cell layers) surfaces. The architecture and metabolism of biofilms confer enhanced resistance to host immune responses, particularly true of antimicrobial tear film, thereby facilitating chronic or recurrent infection. The stages of biofilm formation are presented in Figure 1.

Moreover, intraocular lenses (IOLs) made of abiotic polymers, such as acrylic materials, have been shown to support biofilm formation by Gram-positive bacteria [23,24,25,26,27,28]. In addition to bacterial pathogens, fungi play an increasingly recognized role in ocular infections, with certain regions reporting them as a leading cause of blindness. *C. albicans* remains the most frequently isolated fungal species from the ocular surface [29]. Its ability to form biofilms contributes to poor treatment outcomes, which are further complicated by suboptimal drug targeting, low tissue penetration, the limited bioavailability of antifungal agents, and intrinsic resistance mechanisms [30,31,32]. When inadequately treated, ocular infections can progress to irreversible structural damage, permanent visual impairment, and, in severe cases, blindness. Although the eye is equipped with several innate defense mechanisms—most notably the continuous flow of antimicrobial tear film—microbial invasion, sustained inflammation, and tissue scarring demand prompt and effective therapeutic intervention [6,33].

## 2. Bacterial Biofilms in Ophthalmic Infections

As mentioned, one of the most formidable challenges in managing ocular infections is the presence of bacterial biofilms—structured communities of microbial cells embedded in a self-produced extracellular matrix (ECM) composed of polysaccharides, proteins, lipids, and extracellular DNA. From a clinical standpoint, biofilms are characterized by marked resistance to antimicrobial agents, evasion of host immune defenses, and an enhanced capacity to colonize both biological tissues and abiotic surfaces, including implanted medical devices such as intraocular and contact lenses [16]. Within biofilms, microbial cells coordinate their behavior through quorum sensing (QS), which is a cell density-dependent signaling system that enables collective and simultaneous responses to environmental stimuli [22].

Biofilms may consist of a single microbial species or be polymicrobial, encompassing both bacteria and fungi, thereby increasing their complexity and resilience. Biofilm architecture is inherently stratified, supporting diverse physiological and metabolic states within the same structure. In the upper parts, microbial cells benefit from adequate oxygen and nutrient availability, supporting active metabolism and rapid replication. In contrast, cells in the inner core and bottom parts experience nutrient limitation and hypoxia, leading to metabolic downregulation or entry into a dormant or an abiotic state. This physiological heterogeneity results in differential susceptibility to antibiotics, particularly those targeting active cellular processes such as transcription and translation [4]. The ECM functions as a defensive shield, impeding phagocytosis, antibody penetration, and antibiotic diffusion, thereby fostering microbial persistence despite pharmacological and immune pressure. The matrix also significantly reduces the local concentration of antimicrobials, further promoting chronic colonization and infection [3]. Importantly, biofilm fragments can detach—either due to mechanical forces or internal signaling via QS—and disseminate through the bloodstream, leading to the formation of secondary infection sites. Once dispersed, microbial cells reattach to new surfaces via nonspecific interactions, such as electrostatic forces between the microbial envelope (e.g., membrane, capsule) and the substrate. Notably, pathogens’ physicochemical surface properties affect their adhesion; for instance, *P. aeruginosa* exhibits a highly hydrophobic surface, whereas *S. aureus* is more hydrophilic [4]. These differences influence specific biomaterials’ likelihood of successful colonization. After initial attachment, the local environment becomes highly heterogeneous. Metabolic byproducts tend to accumulate in the central regions of the biofilm, while nutrients are more accessible at the periphery. These biochemical gradients drive functional differentiation, triggering ECM production once a threshold cell density is reached. The matrix components may be secreted by viable cells or released via autolysis, further stabilizing the biofilm architecture [9]. It is now widely recognized that most pathogenic microorganisms exist predominantly in biofilm form, including those isolated from the conjunctival surface of the eye and from intraocular and contact lens-associated infections [9,15,20,21,34,35,36].

This paradigm has important therapeutic implications, as biofilm-embedded cells are markedly less susceptible to antimicrobial agents and host defenses. The clinical relevance of biofilms is perhaps most evident in bacterial keratitis, with an estimated 30,000 cases annually in the United States and over 100,000 worldwide [37]. Among the most significant risk factors is mechanical trauma related to contact lens use [38]. Prolonged lens wear compromises the protective function of the corneal epithelium, increasing its susceptibility to microbial invasion. Microtrauma resulting from debris accumulation, blinking-induced friction, and localized pressure facilitates bacterial access to deeper stromal layers, where biofilm formation can perpetuate persistent and treatment-resistant infection.

## 3. Biofilms in Device-Associated Ocular Infections

Importantly, ocular infections may not only originate from microorganisms residing on the ocular surface but also from those colonizing contact lens materials or their storage cases. Biofilms that develop on contact lenses typically harbor both endo- and exogenous microbial populations. The pathogens that are most frequently implicated are Gram-positive *Staphylococcus* spp. and Gram-negative *P. aeruginosa* [21]. Once established, the biofilm becomes highly resistant/tolerant to tear film defenses and may release endo- and exotoxins that damage the corneal epithelium, facilitating further microbial invasion [39,40,41]. In the United States, fungal keratitis remains considerably less prevalent than its bacterial counterpart; however, contact lens wear is a major predisposing factor for both. A significant proportion of fungal keratitis cases is attributed to *Fusarium* spp., while the protozoan *Acanthamoeba* is responsible for a rare but aggressive form, which is also frequently linked to contact lens use [42,43,44,45]. Another condition associated with biofilm-forming pathogens is infectious crystalline keratopathy (ICK), a slowly progressive corneal infection marked by branching crystalline opacities and minimal inflammation. ICK commonly develops after corneal surgery, especially penetrating keratoplasty, and is associated with *Viridans* group streptococci (VGS), various fungal species, and *Acanthamoeba*.

The involvement of biofilms in ICK has been corroborated via electron microscopy, which has revealed the presence of *C. albicans* and bacterial cells embedded within affected corneal tissues [46,47,48,49]. The risk of keratitis in contact lens users is influenced not only by microbial contamination but also by the physical properties of the lens material and wearing schedules. Epidemiological data indicate that soft contact lenses worn during the day carry a higher infection risk than rigid gas-permeable lenses. Notably, wearing soft lenses overnight, particularly silicone hydrogel and daily disposable types, has been strongly associated with increased keratitis incidence, despite offering enhanced user comfort compared to older materials [50]. Biofilm formation is also a critical concern in cataract surgery, particularly in relation to the implantation of intraocular lenses (IOLs). Designing IOLs with optimized surface properties has become a key strategy in minimizing postoperative infection risk. Commonly used materials include polymethylmethacrylate (PMMA), acrylic, hydrogel, and silicone, each differing in mechanical stiffness and surface hydrophilicity or hydrophobicity, which affects their susceptibility to microbial adhesion [24,27]. In a study by Fazly et al., hydrophobic IOL materials were shown to attract fewer bacteria and bacterial products than their hydrophilic counterparts [51]. More specifically, acrylic lenses were found to be highly susceptible to Gram-positive biofilm formation, while being less prone to colonization by *P. aeruginosa* compared to PMMA and silicone lenses. These findings reflect the differential surface polarity of the pathogens: *P. aeruginosa* is highly hydrophobic, whereas *Staphylococcus* species are predominantly hydrophilic. Although *P. aeruginosa* is not a leading cause of endophthalmitis following uncomplicated cataract surgery, its presence has been documented in clinical cases, often due to environmental contamination, including intraoperative fluids and phacoemulsification machine tubing [52].

## 4. Conventional Antibiotic Therapy in Ocular Infections

Standard treatment for bacterial ocular infections typically involves the use of broad-spectrum antibiotics targeting both Gram-positive and -negative organisms. For optimal efficacy, these agents must achieve sufficient tissue penetration through the conjunctiva and cornea to reach therapeutic concentrations in intraocular tissues. In clinical ophthalmology, bactericidal agents are generally preferred over bacteriostatic ones, as they offer faster microbial clearance and reduce the likelihood of resistance development. The most prescribed antibiotic classes include aminoglycosides and fluoroquinolones, both of which exhibit strong activity against a wide range of ocular pathogens. These agents are typically administered topically in the form of eye drops or ointments, although systemic therapy may be initiated in severe or deep-seated infections, such as endophthalmitis [53,54,55,56,57,58]. Groups of antibiotics used in the conventional therapy in ocular infections and their mechanisms of action are presented in Table 1 and Table 2.

## 5. Challenges of Local Treatment in Ophthalmic Infections

The unique anatomical and physiological barriers of the eye pose considerable challenges when it comes to effective drug delivery. These barriers limit the penetration of systemically administered agents, rendering topical administration as the primary route for treating most anterior segment ocular infections. While general pharmacokinetic principles—absorption, distribution, metabolism, and elimination—apply to ophthalmic drugs, the administration route critically determines the extent of bioavailability within ocular compartments. When a drug is introduced into the conjunctival sac, its absorption is influenced by a multitude of factors, including residence time, tear film composition, elimination through the nasolacrimal drainage system, binding to tear proteins, enzymatic degradation, and penetration across the corneal and conjunctival epithelium. Topically applied medications may also be systemically absorbed, either through local pathways (e.g., transcorneal or transconjunctival diffusion) or via nasal mucosa uptake, thereby potentially reaching systemic circulation. In fact, all ophthalmic drugs carry the potential for systemic exposure, which may result in unintended effects. The most common adverse effects of these drugs arise from local toxicity or hypersensitivity reactions, manifesting as irritation or inflammation of the cornea, conjunctiva, periorbital skin, or nasal mucosa. One of the most significant limitations of topical therapy is its short ocular surface contact time. The tear film is renewed every 2–3 min, while standard eye drops typically remain on the ocular surface for only 15–30 s. As a result, less than 5% of the administered drug reaches intraocular tissues in therapeutically relevant concentrations. To improve ocular bioavailability, various strategies have been developed, including the incorporation of cyclodextrins, viscosity-enhancing agents, and formulation pH optimization [59,60]. The physiological pH of the tear film ranges from 7.3 to 7.7, though this briefly drops upon awakening due to the accumulation of acidic metabolic byproducts during eyelid closure. Eye drops with a pH between 6.0 and 9.0 are generally well tolerated. Outside this range, however, ocular discomfort and reflex tearing may occur, resulting in dilution and reduced therapeutic efficacy. Another critical parameter is osmolarity, which depends on the concentration of dissolved solutes. Excipients in the formulation can alter osmolarity, thereby influencing viscosity, droplet size, and dose delivery dynamics. Deviations from the intended osmolarity may result in under- or overdosing, diminishing therapeutic effectiveness and increasing the risk of local or systemic side effects [61]. Further challenges emerge in multidrug regimens, which are frequently employed in managing ocular inflammation or infection. Short dosing intervals can lead to inter-drug washout, where one formulation physically displaces another, reducing the efficacy of both agents. In contrast, ophthalmic ointments, while advantageous due to their prolonged retention time, are typically reserved for nighttime use because of their tendency to cause transient visual impairment. As a result, their use is contraindicated in patients engaged in activities requiring visual acuity, such as driving or operating machinery [61,62]. Collectively, these limitations underscore the pharmacological and practical challenges associated with conventional topical treatments and highlight the need for innovative strategies—including advanced delivery systems and novel therapeutic agents—to enhance treatment outcomes in ocular infections.

## 6. Emerging Strategies in Ophthalmic Infection Treatment

The rising prevalence of antibiotic resistance in ophthalmology represents an urgent and expanding global health concern. Large-scale surveillance studies, such as the ARMOR study (2009–2016, USA), have documented a progressive increase in antibiotic-resistant ocular pathogens, a trend that is also observed in European countries, including Poland, the authors’ country of origin [56,63]. Contributing factors include prolonged or repeated exposure to subtherapeutic concentrations of antibiotics or chemotherapeutic agents, which fosters the selection of resistant microbial populations. A particularly challenging aspect of ocular infections is the formation of biofilms by both bacterial and fungal pathogens. Biofilms profoundly impair the efficacy of conventional antibiotics and antifungals and compromise the function of medical devices, such as contact lenses and intraocular implants. The extracellular matrix (ECM) of biofilms acts as a physical and biochemical barrier, reducing drug penetration and shielding embedded pathogens from immune surveillance. In response to this escalating therapeutic challenge, increasing attention has been paid to natural, plant-derived compounds with antibiofilm activity.

In recent years, significant research efforts have been dedicated to exploring the therapeutic potential of polyphenolic compounds, essential oils, terpenoids, alkaloids, and lectins. These phytochemicals not only inhibit biofilm formation but also possess the ability to disrupt established biofilms, making them attractive candidates for treating recalcitrant ocular infections. Importantly, the mechanisms of action of plant-derived antimicrobials are often multifaceted and distinct from those of conventional antibiotics. Whereas synthetic antibiotics typically target specific cellular processes—such as cell wall synthesis or protein translation, many phytochemicals exert multi-targeted effects, simultaneously compromising biofilm structure, quorum sensing networks, and microbial adhesion mechanisms. This broad-spectrum and synergistic mode of action holds significant therapeutic promise in biofilm-associated ocular infections, where monotherapy with conventional agents often fails to achieve complete resolution [64,65,66].

## 7. Plant-Derived Antimicrobials in Ocular Infection Management

The growing popularity of natural therapies over the past several decades has catalyzed intensive research into designing effective delivery systems for plant-derived bioactive compounds, particularly in the context of vision-threatening ocular diseases. Phytochemicals have long been appreciated for their safety, efficacy, cultural acceptability, and less frequent side effects compared to synthetic drugs. Today, an increasing number of natural extracts are being incorporated as active ingredients in modern ophthalmic pharmaceutical formulations [66]. Crucially, plant-derived antimicrobials often act via mechanisms fundamentally distinct from classical antibiotics, substantially lowering the likelihood of resistance development. This makes them attractive alternatives or adjuncts in managing biofilm-mediated ocular infections, particularly in cases involving multidrug-resistant organisms. Several major classes of phytochemicals have demonstrated significant antimicrobial and antibiofilm activity in preclinical studies and are currently under active investigation. Among the best-characterized polyphenolics are curcumin (*Curcuma longa* L., Zingiberaceae) and resveratrol (from grapes; *Vitis vinifera* L., Vitaceae), both of which exhibit potent antimicrobial and antibiofilm effects. Curcumin has been shown to disrupt bacterial cell membranes and inhibit quorum sensing, the cell-to-cell signaling mechanism essential for biofilm formation in many resistant bacterial strains. Resveratrol, meanwhile, has demonstrated efficacy against *S. aureus* biofilms, positioning it as a promising candidate for applications such as ocular wound healing and medical device coatings [67,68,69,70,71,72,73]. Comparison of plant-derived antimicrobial compounds is presented in Table 3.

### 7.1. Resveratrol

**Figure 2:**
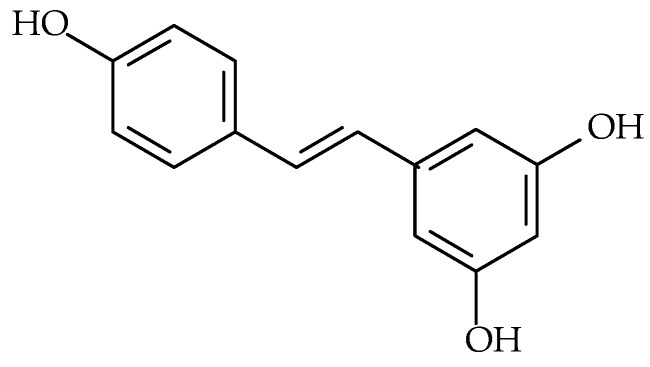
Chemical structure of resveratrol.

Resveratrol (syn.: 3,5,4′-trihydroxy-trans-stilbene, 5-[(E)-2-(4-hydroxyphenyl)-ethenyl]-benzene-1,3-diol, molecular formula: C_14_H_12_O_3_, molecular weight: 228.24 g/mol) (Figure 2), a non-flavonoid phenolic compound belonging to the stilbene subclass, has attracted significant scientific interest due to its broad spectrum of biological activities, including antioxidant, anti-inflammatory, antiapoptotic, and antiangiogenic effects [85,86].

Its therapeutic potential has been explored in various systemic conditions, such as cancer, cardiovascular and neurological disorders, and diabetes, as well as in ocular diseases including cataracts, glaucoma, and diabetic retinopathy [87]. Despite this favorable pharmacological profile, resveratrol’s clinical applicability is significantly hindered by its low oral bioavailability, which is primarily due to its poor aqueous solubility, rapid metabolism, and short systemic half-life [88]. These limitations have driven the development of advanced delivery platforms, including nanoparticles (NPs), micelles, liposomes, and ocular inserts, with the aim of improving tissue penetration, stability, and sustaining therapeutic effects [89]. In a notable in vivo study by Dong et al., gold nanoparticles (AuNPs) coated with resveratrol were evaluated in a streptozotocin-induced diabetic retinopathy model using male Wistar rats [90]. Daily administration of resveratrol-loaded AuNPs (300 mg/kg/day) significantly attenuated retinal vascular leakage, histological retinal damage, and the expression of proinflammatory cytokines (IL-6, IL-1β) and adhesion molecules (ICAM-1, VCAM-1). Cytokine levels were reduced by more than 30% compared to untreated diabetic controls, suggesting that nanocarrier-enhanced resveratrol may provide neurovascular protection in diabetic ocular pathology [90]. From a microbiological standpoint, resveratrol exhibits more pronounced antifungal than antibacterial activity. At 10–20 µg/mL, it has demonstrated inhibitory effects against dermatophytes such as *Trichophyton mentagrophytes*, *T. tonsurans*, *T. rubrum*, *Epidermophyton floccosum*, and *Microsporum gypseum* at concentrations ranging from 25 to 50 µg/mL, as well as against yeasts including *Candida albicans*, *Saccharomyces cerevisiae*, and *Trichosporon beigelii* [91,92]. However, conflicting results have been reported, with several studies observing no significant antifungal activity against *C. albicans* [93,94,95]. Resveratrol has also been shown to inhibit the plant fungal pathogen *Botrytis cinerea* at concentrations of 60–140 µg/mL, primarily by suppressing conidial germination and mycelial growth [95]. In contrast, its antibacterial effects are generally observed at higher concentrations (≥100 µg/mL). Among the Gram-positive bacteria, *S. aureus*, *Enterococcus faecalis*, and *Streptococcus pyogenes* exhibit minimum inhibitory concentrations (MICs) in the range of 100–200 µg/mL [91,93,96]. Gram-negative organisms, including *Escherichia coli*, *Klebsiella pneumoniae*, *Salmonella typhimurium*, and *Pseudomonas aeruginosa*, are typically less susceptible, requiring MICs exceeding 200 µg/mL [91,96]. This disparity is likely attributable to the limited outer membrane permeability or active efflux pump mechanisms present in Gram-negative species.

### 7.2. Curcumin

**Figure 3:**
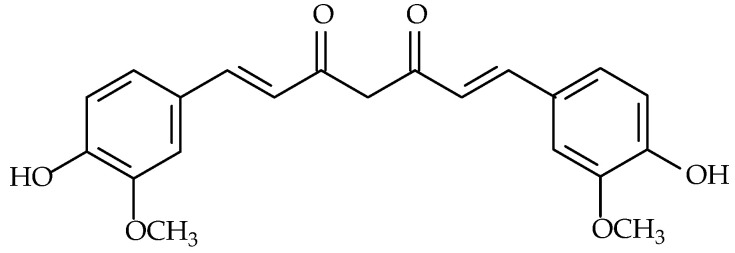
Chemical structure of curcumin.

Curcumin (syn.: (1E,6E)-1,7-bis(4-hydroxy-3-methoxyphenyl)-hepta-1,6-diene-3,5-dione, diferuloylmethane; molecular formula: C_21_H_20_O_6_, molecular weight: 368.4 g/mol) (Figure 3), the principal curcuminoid of turmeric (*Curcuma longa* L., Zingiberaceae, roots), has been extensively investigated for its pleiotropic health benefits, including applications in aging, diabetes, depression, chronic inflammation, and oncology [97,98,99,100,101,102].

Despite its promising biological profile, curcumin’s clinical translation is limited by several pharmacokinetic drawbacks, such as rapid metabolism, poor aqueous solubility, chemical instability, and low cellular permeability. To overcome these barriers, innovative formulation strategies have been explored [103]. One such approach involves the use of Calix [4] arene derivatives with lipid-mimetic dodecyl chains, which self-assemble with curcumin, enhancing its solubility nearly 9000-fold and inhibiting the nuclear translocation of inflammation-associated transcription factors [104]. Similarly, Sai et al. developed an in situ gelling system based on micelles (PEG-DSPE/Solutol HS15) and gellan gum, which improved ocular retention and reduced curcumin degradation to 1.4% (vs. 34% for free curcumin) in rabbits, while causing no ocular irritation [105]. Another strategy involved a thermoresponsive gel composed of Pluronic F127/F68 and curcumin-loaded albumin nanoparticles, which enhanced the curcumin concentration in the aqueous humor 5.6-fold without inducing ocular toxicity [106]. Curcumin demonstrates broad-spectrum antimicrobial activity, including against multidrug-resistant (MDR) pathogens such as MRSA and polymyxin-resistant *K. pneumoniae*. Reported MIC values vary widely; for MRSA isolates, inhibition occurs at 125–500 µg/mL, while MDR *Acinetobacter baumannii*, *P. aeruginosa*, and *K. pneumoniae* are inhibited at 128–512 µg/mL. These variations are likely attributable to solvent choice (e.g., DMSO, ethanol, water), methodological differences, and curcumin purity [107,108].

### 7.3. Abietic Acid

**Figure 4:**
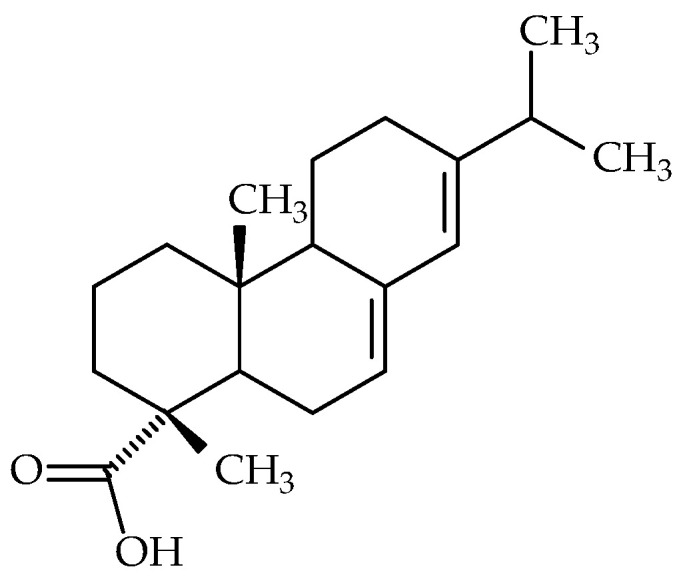
Chemical structure of abietic acid.

Abietic acid (syn.: (1R,4aR,4bR,10aR)-1,4a-dimethyl-7-propan-2-yl-2,3,4,4b,5,6,10,10a -octahydrophenanthrene-1-carboxylic acid, ABA; molecular formula: C_20_H_30_O_2_, molecular weight: 302.5 g/mol) (Figure 4), which is traditionally used in Korean and Japanese medicine, is a diterpene resin acid and the principal component of pine resin (Pini resina).

Pini resina is a natural resin obtained from plants belonging to the Pinaceae family. It is commercially produced from selected *Pinus* species, including *P. palustris* Mill., *P. pinaster* Ait., *P. sylvestris* L., *P. laricio* Poiret, *P. longifolia* Roxb., *P. densiflora* Siebold et Zucc., and *P. thunbergii* Parlatore. It exhibits a broad pharmacological profile, including anti-inflammatory, antiallergic, antimicrobial, antiulcer, and angiogenic properties [84,109]. In studies conducted by our group, ABA was evaluated for its antibiofilm activity against *S. aureus, P. aeruginosa*, and *C. albicans*. At 512 µg/mL, ABA inhibited bacterial biofilms by approximately 50% and fungal equivalents by 20%. Increasing the concentration to 1024 µg/mL enhanced these effects: inhibition reached ~75% for *S. aureus*, ~65% for *P. aeruginosa*, and ~60% for *C. albicans*. Time-dependent analyses indicated the faster disruption of bacterial biofilms (15–30 min) compared to fungal equivalents, which required up to 24 h. Cytotoxicity testing using fibroblast and keratinocyte cell lines demonstrated that ABA is non-toxic up to 256 µg/mL, supporting its potential for further in vivo development.

In the *Galleria mellonella* larval model, ABA significantly improved larval survival in infections with *S. aureus*, confirming its bactericidal activity. Notably, ABA exhibited differential activity against Gram-positive and -negative bacteria, likely due to differences in cell wall composition, biofilm adherence, and efflux pump mechanisms. Additionally, synergistic effects were observed with conventional antibiotics, which were possibly mediated by membrane destabilization and efflux system inhibition. Overall, these findings position ABA as a promising candidate for managing biofilm-associated, antibiotic-resistant ocular infections, particularly when delivered via optimized topical formulations [110]. In the broader context of non-ocular biofilm-related infections, our preliminary studies also suggest its utility beyond ophthalmology [111].

### 7.4. Essential Oils and Their Active Constituents

Essential oils (EOs) have long been used in traditional medicine and are increasingly being investigated for their antimicrobial potential in ocular infections. One notable example is eyebright (*Euphrasia rostkoviana* Hayne, Orobanchaceae, aerial parts), a medicinal plant traditionally used for conjunctivitis and blepharitis, which are often of bacterial origin. In a study by Pavel Nový et al., the chemical composition and antimicrobial activity of eyebright essential oil were examined against pathogens commonly implicated in ocular infections, including *E. faecalis*, *E. coli*, *K. pneumoniae*, *S. aureus*, *S. epidermidis*, *P. aeruginosa*, and *C. albicans*. Gas chromatography–mass spectrometry (GC–MS) analysis identified over 70 constituents, with n-hexadecanoic acid (18.47%), thymol (7.97%), myristic acid (4.71%), linalool (4.65%), and anethole (4.09%) among the most abundant. The essential oil exhibited antimicrobial activity against all tested organisms except *P. aeruginosa*, with the strongest inhibition observed for Gram-positive species, exhibiting MIC values around 512 µg/mL [112]. These findings underscore the potential role of EOs in developing natural, broad-spectrum therapies for superficial ocular infections, particularly in the context of rising antimicrobial resistance.

**Figure 5:**
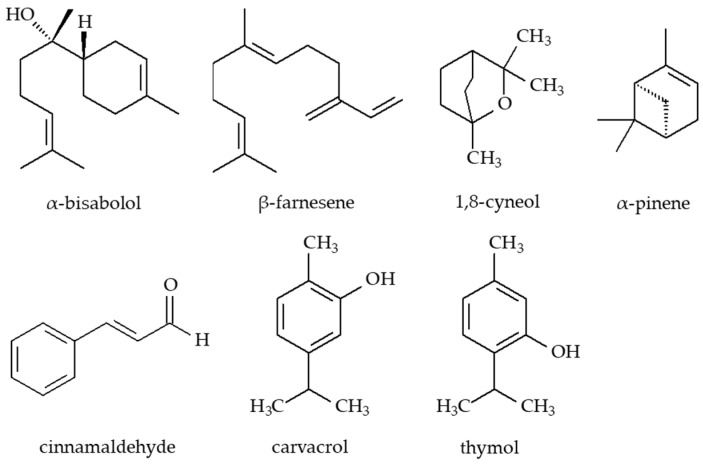
Chemical structures of the main constituents of essential oils.

Microbial keratitis is a rapidly progressing ocular infection requiring urgent antimicrobial intervention. As resistance to synthetic agents continues to escalate, interest in plant-derived alternatives, particularly essential oils (EOs), has grown considerably. These natural products exhibit broad-spectrum biological activities, including antibacterial, antifungal, antiviral, and antioxidant properties, making them attractive candidates for ocular therapeutic development [113]. In a study by El-Badry et al. [114], the antimicrobial efficacy of 15 essential oils against resistant strains of *Aspergillus niger* and *S. aureus* was evaluated using agar diffusion assays. Chamomile essential oil (*Chamomilla recutita* L., Asteraceae, flowers) showed the strongest antifungal activity against *A. niger*, while rosemary essential oil (*Rosmarinus officinalis* L., Lamiaceae, leaves) exhibited potent antibacterial effects against *S. aureus*, findings consistent with previous reports [115,116,117,118]. Biochemical identification (including VITEK 2C testing) confirmed the microbial isolates, while FT-IR spectroscopy demonstrated the absence of toxic functional groups such as cyanide (C≡N) or acetylene (C≡C). Active moieties such as aldehydes (-CHO) and aliphatic double bonds (C=C) were detected, supporting the biological activity of these oils. Minimum inhibitory concentration (MIC) analysis revealed that 60% (*v*/*v*) chamomile oil effectively inhibited *A. niger*, whereas 40% (*v*/*v*) rosemary oil was active against *S. aureus*. The high susceptibility of *S. aureus* is likely attributable to the simpler cell wall architecture of Gram-positive bacteria, which facilitates easier penetration by lipophilic phytochemicals [119]. Ultrastructural analysis using transmission electron microscopy (TEM) revealed severe damage in both fungi and bacteria exposed to essential oils. In *A. niger*, notable changes included cell wall degradation, organelle disruption, cytoplasmic leakage, and protein aggregation [120]. In *S. aureus*, rosemary oil induced cell membrane shrinkage, cell wall rupture, and intracellular protein clumping [121]. Further validation was provided using a murine model, where a histological analysis of corneal tissues treated with chamomile and rosemary oils showed a well-preserved architecture and only mild inflammatory infiltrates. In contrast, corneas treated with itraconazole exhibited severe tissue destruction, while those exposed to gatifloxacin showed moderate inflammatory responses [114]. Overall, these findings highlight the antimicrobial efficacy, mechanistic complexity, and favorable safety profile of selected essential oils, particularly chamomile and rosemary. Their ability to destabilize microbial membranes, combined with their low cytotoxicity, positions them as viable, natural alternatives to conventional topical agents in managing corneal infections and other ocular surface pathologies [110,122]. All chemical structures of the active components of essential oils are presented in Figure 5.

A summary of the probable mechanism of action of the compounds discussed is provided in Table 4.

## 8. Limitations: A Critical Analysis

Despite the promising antimicrobial potential of plant-derived agents in ophthalmology, several substantial challenges emerge when translating phytotherapeutics into clinical practice. One of the primary obstacles is the inherent variability in the chemical composition of plant extracts, which is influenced by numerous environmental and genetic factors, including plant species, growth conditions, harvesting time, and extraction methods [123]. This variability complicates the standardization of herbal preparations and hinders reproducibility across different batches, ultimately affecting the consistency of therapeutic outcomes. Studies have shown that concentrations of bioactive constituents may vary significantly, even within the same nominal product; this makes dose determination, pharmacokinetic assessment, and inter-study comparisons unreliable [124]. In addition, concerns remain regarding the ocular safety profile of many phytochemicals. Certain plant extracts may exert irritation or even cytotoxic effects on the delicate structures of the eye, particularly when used in non-standardized concentrations or formulations [125]. There are documented cases of severe ocular surface damage following the traditional application of herbal remedies, some of which have resulted in visual deterioration and irreversible complications [126]. Moreover, hypersensitivity reactions have been reported; for example, Chamomilla eye washes have been associated with allergic conjunctivitis due to pollen allergens [127]. Non-sterile or contaminated herbal products may also introduce pathogens or toxins, exacerbating existing infections or triggering new ones [128]. These adverse effects underscore the need for rigorous toxicological screening and ocular safety evaluations prior to clinical application. Another key limitation is the poor bioavailability of many phytochemicals. Most plant-derived secondary metabolites exhibit low aqueous solubility, limited corneal permeability, and rapid systemic clearance, all of which hinder their accumulation at therapeutic levels in ocular tissues [129]. For example, although curcumin demonstrates significant anti-inflammatory and antimicrobial activity, its low solubility and poor absorption drastically limit its clinical utility, unless delivered via advanced drug delivery systems such as liposomes, cyclodextrins, or nanoparticles [130]. While these delivery platforms show promise in enhancing tissue penetration and prolonging ocular residence time, most remain in the preclinical stage, with limited translation into commercially available ophthalmic formulations [131]. Equally important is the lack of robust clinical evidence supporting the efficacy and safety of plant-based therapies in ophthalmology. Although numerous in vitro and in vivo studies have reported the antimicrobial activity of herbal compounds against ocular pathogens, high-quality randomized controlled trials (RCTs) involving human subjects remain scarce [73]. A recent review of traditional ocular remedies documented the use of over 60 medicinal plants for various eye conditions, yet only one plant had been formally evaluated in a clinical setting [132]. The paucity of clinical validation limits the incorporation of these therapies into evidence-based treatment guidelines. Similar gaps have been observed in other medical specialties, such as dermatology and dentistry, where the promising in vitro effects of phytochemicals have not consistently been translated into clinical adaptation [133]. Challenges in study design—such as the use of placebo controls, extract standardization, and funding constraints—further impede the generation of strong clinical evidence [134]. Finally, regulatory uncertainties represent a critical barrier to the integration of phytoconstituents into conventional ophthalmic care. Herbal medicines are often classified differently across jurisdictions, resulting in fragmented and inconsistent regulatory pathways [135]. In the European Union, for instance, simplified registration procedures allow some products to enter the market as traditional herbal medicinal products without the need for clinical trials, provided there is sufficient evidence of long-standing safe use [136]. While this facilitates access, it may also raise concerns about the adequacy of efficacy and safety assessments. In many countries, herbal products are marketed as dietary supplements or cosmetic formulations, bypassing the rigorous quality and safety standards required for pharmaceutical drugs [137]. These regulatory discrepancies contribute to limited oversight, inconsistent product quality, and skepticism among clinicians regarding the reliability of phytotherapeutics in ocular care [138]. Therefore, the significant barriers to the clinical implementation of plant-based antimicrobial agents in ophthalmology include their chemical variability, the lack of standardization, the potential for adverse effects, poor bioavailability, limited clinical evidence, and regulatory ambiguity. Other strategies, including the use of metallic nanoparticles, have, thus, also been developed [139]. Addressing these challenges will require comprehensive pharmacological, toxicological, and clinical investigations, as well as the establishment of robust quality control frameworks. Only then can the therapeutic potential of phytochemicals be safely and effectively harnessed for treating ocular infections.

## 9. Conclusions

The escalating crisis of antibiotic resistance has emerged as one of the most urgent public health challenges of the 21st century. The search for alternative therapeutic strategies has intensified in response to the growing limitations of conventional antimicrobials. Among the most promising approaches is the use of plant-derived compounds, which have demonstrated broad-spectrum antimicrobial activity, including efficacy against multidrug-resistant pathogens. Many of these natural agents, which have long been utilized in traditional medicine, are now gaining renewed scientific validation as viable and sustainable alternatives to synthetic drugs. Plant-based antimicrobials offer unique advantages due to the diversity, structural complexity, and multifunctionality of their bioactive constituents. These compounds represent a largely untapped reservoir of pharmacological potential and may serve as scaffolds for the development of novel anti-infective therapies. Their ability to target multiple microbial pathways, including biofilm formation, quorum sensing, and membrane integrity—offers a multifaceted mechanism of action that could complement or enhance existing treatment paradigms. When appropriately formulated, phytochemicals may significantly contribute to overcoming the current limitations in managing ocular surface infections. However, despite their promise, several critical barriers must be addressed before plant-derived agents can be fully integrated into clinical ophthalmology. Standardization and quality control remain major challenges due to the inherent chemical variability of botanical extracts, which is influenced by environmental, genetic, and processing factors. Additionally, the poor aqueous solubility and low ocular bioavailability of many phytochemicals necessitate the use of advanced delivery systems—such as nanoparticles, cyclodextrins, or liposomes—to ensure therapeutic efficacy and tissue penetration. Equally important is the need for robust clinical evidence. While numerous in vitro and pre-clinical studies support the antimicrobial activity of phytotherapeutics, well-designed randomized controlled trials remain scarce. Without strong clinical validation, their acceptance by regulatory bodies and integration into evidence-based practice will remain limited. Furthermore, regulatory frameworks governing the approval of plant-based therapeutics are often fragmented or inconsistent, posing additional obstacles to commercialization and clinical implementation. Plant-derived antimicrobials represent a promising and underutilized resource in global efforts to combat antimicrobial resistance, including in the context of biofilm-associated ocular infections. Their successful translation into clinical practice will depend on rigorous pharmacological and toxicological evaluation, formulation innovation, clinical validation, and the development of coherent regulatory pathways. Therefore, future perspectives should focus on the rational design of standardized, well-characterized phytocompounds with proven efficacy against biofilm-forming ocular pathogens. Emphasis should be placed on developing advanced delivery systems—such as nanoparticles, micelles, or in situ gels—to overcome solubility and bioavailability limitations. Integrating plant-derived molecules into combination therapies with conventional antibiotics may also enhance therapeutic outcomes while minimizing resistance development. Finally, well-designed clinical trials are essential to validate preclinical findings and support regulatory approval for safe ophthalmic use. With continued interdisciplinary research and strategic investment, phytochemicals may soon play a transformative role in shaping the future of infectious disease therapy in ophthalmology and beyond.

## Figures and Tables

**Table 1:** Mechanisms of action of aminoglycosides and fluoroquinolones [53,54,55,56,57,58].

Group of Antibiotics	Mechanism of Action	Spectrum of Activity
Aminoglycosides	Passive binding: the antibiotic binds to Ca^2+^ and Mg^2+^ receptors in the lipopolysaccharide layer of the bacterial membrane. This binding is proportional to the antibiotic concentration and is inhibited by Ca^2+^ and Mg^2+^ ions. Active transport: the drug enters the cell via electrostatic forces, damaging the cytoplasmic membrane and causing nutrient leakage. This transport requires oxygen and energy, making aminoglycosides only effective against aerobic bacteria. Ribosomal binding and protein synthesis inhibition: binds to the 30S ribosomal subunit, blocking translation and leading to bacterial cell death.	Strong antibacterial activity: effective against Gram-negative bacteria (*Haemophilus*, *Pseudomonas*, *Enterobacteriaceae*) and *Staphylococcus aureus* (except some MRSA strains). Limited activity against *Streptococci*.
Fluoroquinolones	Inhibition of DNA synthesis: Blocks type II topoisomerases, enzymes responsible for cutting both DNA strands: DNA gyrase: enzyme responsible for DNA supercoiling and spatial isomer formation. Topoisomerase IV: enzyme that separates DNA strands after replication. Inhibiting these enzymes leads to DNA damage, structural relaxation, and bacterial cell death.	Highly effective against Gram-negative bacteria (*Haemophilus*, *Salmonella*, *Neisseria*, *Pseudomonas*, *Enterobacteriaceae*). Fourth-generation fluoroquinolones (e.g., gatifloxacin, moxifloxacin, besifloxacin) are also effective against Gram-positive bacteria.

**Table 2:** Antibiotic groups and their activity.

Group of Antibiotics	Exemplary Drugs	Spectrum of Activity
Aminoglycosides	Streptomycin, gentamicin, kanamycin, tobramycin, neomycin, amikacin, sisomicin, netilmicin	-Enterobacterales: *Escherichia coli*, *Klebsiella*, *Enterobacter*, *Proteus* -*Pseudomonas* (including *Pseudomonas aeruginosa*) -*Haemophilus* spp. -*Brucella* spp. -*Pasteurella* spp. -*Mycobacterium tuberculosis* -*Staphylococci* (including *S. aureus*) -Resistance in some strains of *S. aureus*, especially methicillin-resistant strains (MRSA).
Fluoroquinolones	Norfloxacin, enoxacin (Generation I). Ciprofloxacin, ofloxacin, lomefloxacin (Generation II). Levofloxacin (Generation III).	Generation I: Gram-negative bacteria: *Haemophilus influenzae*, *Moraxella*, *Neisseria*, *Chlamydia* spp. Generation II: Gram-negative bacteria: *Haemophilus*, *Pseudomonas*, *Salmonella*, *Neisseria*, *Moraxella* Generation III: Gram-negative bacteria: *Pseudomonas*, *Neisseria*, *Haemophilus*; Gram-positive bacteria: *S. aureus*, *Streptococcus pneumoniae*, *S. pyogenes* -Poor sensitivity to anaerobic bacteria. -Inherited resistance in some strains of *P. aeruginosa*.

**Table 3:** Comparison of plant-derived antimicrobial compounds.

Type of Compound	Plant Extracts (Phytoconstituents)	Mechanism and Activity	Refs.
Polyphenolics (curcuminoids, stilbenes)	Turmeric (curcumin) Grapes (resveratrol)	Curcumin disrupts the bacterial cell membrane and inhibits quorum sensing, a key mechanism for biofilm formation in resistant bacteria. Resveratrol has been tested and showed efficacy against *Staphylococcus aureus* biofilms.	[67,68,69,70,71,72]
Essential oils	Eyebright (thymol) Chamomile (α-bisabolol and its oxides A and B, β-farnesene) Rosemary (1,8-cyneole, α-pinene) Oregano (carvacrol) Thyme (thymol) Cinnamon (cinnamaldehyde)	Disrupt bacterial membranes and metabolic routes. Oregano has efficacy against multidrug-resistant *Escherichia coli* and *Pseudomonas aeruginosa.*	[74,75,76,77]
Alkaloids	Berberis (berberine)	Effective against Gram-positive bacteria, including MRSA. Interferes with bacterial DNA and cell wall synthesis; distinct mode of action compared to conventional antibiotics.	[75,78]
Flavonoids	Green tea (catechins) Onions (quercetin)	Inhibit bacterial enzymes and cell walls. Catechins show synergy with conventional antibiotics, enhancing their action against resistant strains.	[79,80,81]
Tannins and Terpenoids	Neem (*Azadirachta indica)*, Eucalyptus (*Eucalyptus globulus*) Abietic acid (derived from pine tree resin)	Exhibit antimicrobial, antifungal, and antiviral activities. Neem extracts are effective against resistant strains of *Helicobacter pylori.*	[71,82,83,84]

**Table 4:** Mechanisms of action for each compound discussed in Section 7.

Type of Compound	Compounds/Plant Extracts	Mechanism of Action	Refs.
Polyphenolics (curcuminoids, stilbenes)	Curcumin (turmeric) Resveratrol (grapes)	Curcumin disrupts the bacterial cell membrane and inhibits quorum sensing, a key mechanism for biofilm formation in resistant bacteria. Resveratrol has been tested and showed efficacy against *Staphylococcus aureus* biofilms	[67,68,69,70,71,72]
Essential oils	α-Bisabolol and its oxides, β-farnesene (chamomile) 1,8-Cyneole, α-Pinene (rosemary) Carvacrol (oregano) Thymol (eyebright, thyme) Cinnamaldehyde (cinnamon)	Disrupt bacterial membranes and metabolic routes. Oregano has efficacy against multidrug-resistant *Escherichia coli* and *Pseudomonas aeruginosa*	[74,75,76,77]
Terpenoids	Abietic acid Conifer resins, mainly from *Pinus* species	Disrupts microbial cell membranes, increases permeability, and causes leakage of intracellular components. Effective against among others *Pseudomonas aeruginosa*, *Staphylococcus aureus*, *Candida albicans*	[110]

## Data Availability

Not applicable.
